# Case Report: Tracheobronchopathia osteochondroplastica with *Pseudomonas aeruginosa* recovered from bronchoalveolar lavage fluid

**DOI:** 10.3389/fmed.2026.1972198

**Published:** 2026-10-21

**Authors:** Jinlin Li, Dongmei Kuang, Daihua Hu, Canguo Xie, Ling Liao, Xin Dong

**Affiliations:** 1 Department of Respiratory Medicine, Suizhou Hospital, Hubei University of Medicine, Suizhou, China; 2 Department of Pathology, Suizhou Hospital, Hubei University of Medicine, Suizhou, China; 3 Department of Medical Imaging, Suizhou Hospital, Hubei University of Medicine, Suizhou, China

**Keywords:** airway stenosis, bronchoalveolar lavage, bronchoscopy, case report, computed tomography, Pseudomonas aeruginosa, tracheobronchopathia osteochondroplastica

## Abstract

**Background:**

Tracheobronchopathia osteochondroplastica (TO) is an uncommon benign disorder characterized by osseous and/or cartilaginous submucosal nodules involving predominantly the anterolateral tracheobronchial walls. This case illustrates multimodal diagnosis and cautious interpretation of concomitant lower-airway bacterial recovery.

**Case presentation:**

A 63-year-old man presented with a 3-month history of cough and scant whitish mucoid sputum. Computed tomography (CT) demonstrated partially calcified anterolateral tracheobronchial nodules with relative posterior-wall sparing, a right-sided patchy pulmonary opacity, and multiple pulmonary nodules. Retrospective airway measurements showed a minimum tracheal luminal area of 179.4 mm^2^ and a minimum diameter of 10.0 mm at the same level. Bronchoscopy revealed hard yellow-white nodules involving the trachea and proximal bronchi; biopsy showed mature lamellar bone, supporting TO. A pooled bilateral bronchoalveolar lavage specimen obtained after empirical piperacillin/tazobactam had been started yielded *Pseudomonas aeruginosa* at >10⁴ CFU/mL, susceptible to piperacillin/tazobactam and levofloxacin. The patient received 5 days of intravenous piperacillin/tazobactam followed by 9 days of oral levofloxacin and airway-clearance treatment, with symptomatic improvement. He completed the oral course without reported adverse effects. At an outpatient visit approximately 3 months after discharge, he was asymptomatic. The treating clinician's comparison of follow-up CT with baseline images showed resolution of the pulmonary opacity, unchanged airway nodules, and stable pulmonary nodules. Repeat cultures were not obtained.

**Conclusion:**

Concordant CT, bronchoscopic, and supportive histopathologic findings establish the diagnostic pattern of TO. Clinical and radiologic improvement supported a concomitant respiratory infection, but the organism's causal role and any causal contribution of TO remained unproven. This distinction supports conservative airway management and continued surveillance without attributing every pulmonary abnormality to TO.

## 1 Introduction

Tracheobronchopathia osteochondroplastica (TO) is an uncommon benign disorder of the central airways characterized by multiple osseous and/or cartilaginous submucosal nodules protruding into the lumen of the trachea and proximal bronchi. Because these lesions arise predominantly along the cartilaginous rings, they typically involve the anterior and lateral walls while relatively sparing the posterior membranous wall. The etiology and pathogenesis of TO remain incompletely understood (1–4).

The epidemiology of TO is difficult to determine because many patients are asymptomatic or are diagnosed incidentally during computed tomography or bronchoscopy performed for another indication. In a retrospective Chinese cohort of 41,600 bronchoscopic examinations, only 22 cases were identified, corresponding to a detection rate of approximately 0.05%; chronic cough and increased sputum production were the most frequent presenting symptoms (2). Other clinical series have shown that TO may present with exertional dyspnea, wheeze, hemoptysis, recurrent lower respiratory tract infection, or no respiratory symptoms at all, and its nonspecific presentation may lead to misdiagnosis as asthma, chronic bronchitis, or other central-airway disorders (1, 3, 5, 6).

Chest computed tomography may demonstrate irregular or beaded nodular thickening, calcification, and variable narrowing of the anterolateral tracheobronchial walls, whereas bronchoscopy directly visualizes the characteristic hard submucosal nodules and relative sparing of the posterior membranous wall (2, 3, 7, 8). Histopathologic examination may show submucosal cartilage, calcification, or mature bone; however, the necessity of biopsy remains debated when the radiologic and bronchoscopic findings are typical. Tissue sampling is particularly valuable in atypical cases or when alternative diagnoses, including tracheobronchial amyloidosis, papillomatosis, relapsing polychondritis, granulomatosis with polyangiitis, or malignancy, must be excluded (3, 8, 9).

Here, we report a 63-year-old man in whom TO was identified during evaluation of chronic cough and a pulmonary opacity. The case integrates CT, bronchoscopy, histopathology, quantitative bronchoalveolar lavage (BAL) culture, and follow-up imaging. Its clinical value lies in distinguishing improvement of a concomitant pulmonary process from the persistence of structural airway lesions, while avoiding an unsupported causal attribution of *Pseudomonas aeruginosa* infection to TO.

## 2 Case description

### 2.1 Patient information and clinical findings

A 63-year-old man was admitted on 8 June 2026 with a 3-month history of intermittent cough and scant, difficult-to-expectorate whitish mucoid sputum. He denied hemoptysis, fever, night sweats, clinically significant dyspnea, chest pain, or unintentional weight loss. Unspecified intravenous treatment at a local facility had provided little relief. Whether that treatment included antibiotics, and their names and duration, could not be established from available records. Chest CT on 30 May 2026 showed pulmonary abnormalities and tracheobronchial wall irregularity, prompting further evaluation.

His history included hypertension treated with irbesartan and sustained-release nifedipine and previous renal cyst surgery. He was a lifelong nonsmoker and worked as a farmer. On focused questioning, he denied asbestos or crystalline silica exposure, stone processing, and construction work during farming off-seasons. He reported no recurrent respiratory infections, chronic rhinosinusitis, tuberculosis, known immunodeficiency, family history of similar airway disease, prolonged intubation, or tracheostomy. There was no history of long-term systemic corticosteroid use, other immunosuppressive therapy, or chemotherapy.

On admission, his temperature was 36.5 °C, pulse rate was 81 beats/min, respiratory rate was 20 breaths/min, and blood pressure was 146/97 mmHg. Breath sounds were coarse bilaterally, with a few crackles over the right lung. The remainder of the clinically relevant physical examination was unremarkable.

### 2.2 Diagnostic assessment

The leukocyte count was 5.27 × 10⁹/L, with 66.6% neutrophils; hemoglobin was 137 g/L, and the erythrocyte sedimentation rate was 19 mm/h. Renal function and aminotransferase levels were within their reference ranges. C-reactive protein and procalcitonin were not measured at admission. HIV antibody testing was negative; serum immunoglobulin concentrations were not measured.

Chest CT on 30 May 2026 showed an ill-defined right-sided patchy pulmonary opacity. Ground-glass nodules in the right middle and lower lobes measured up to approximately 8 × 6 mm; scattered bilateral solid nodules measured up to approximately 10 × 8 mm in the right lower lobe. The trachea and proximal bronchi showed partially calcified anterolateral nodular wall thickening with intraluminal protrusion and relative posterior membranous-wall sparing (Figure 1). Review showed no bronchiectasis, emphysema, mucus plugging, atelectasis, interstitial lung disease, cavitation, or CT features suggesting aspiration. Retrospective airway analysis using reformations perpendicular to the tracheal axis identified a minimum luminal cross-sectional area of 179.4 mm^2^, with a minimum diameter (Dmin) of 10.0 mm at that level. Because the abnormality involved a long segment without an unequivocally normal reference segment, percentage stenosis was not calculated.

**Figure 1:**
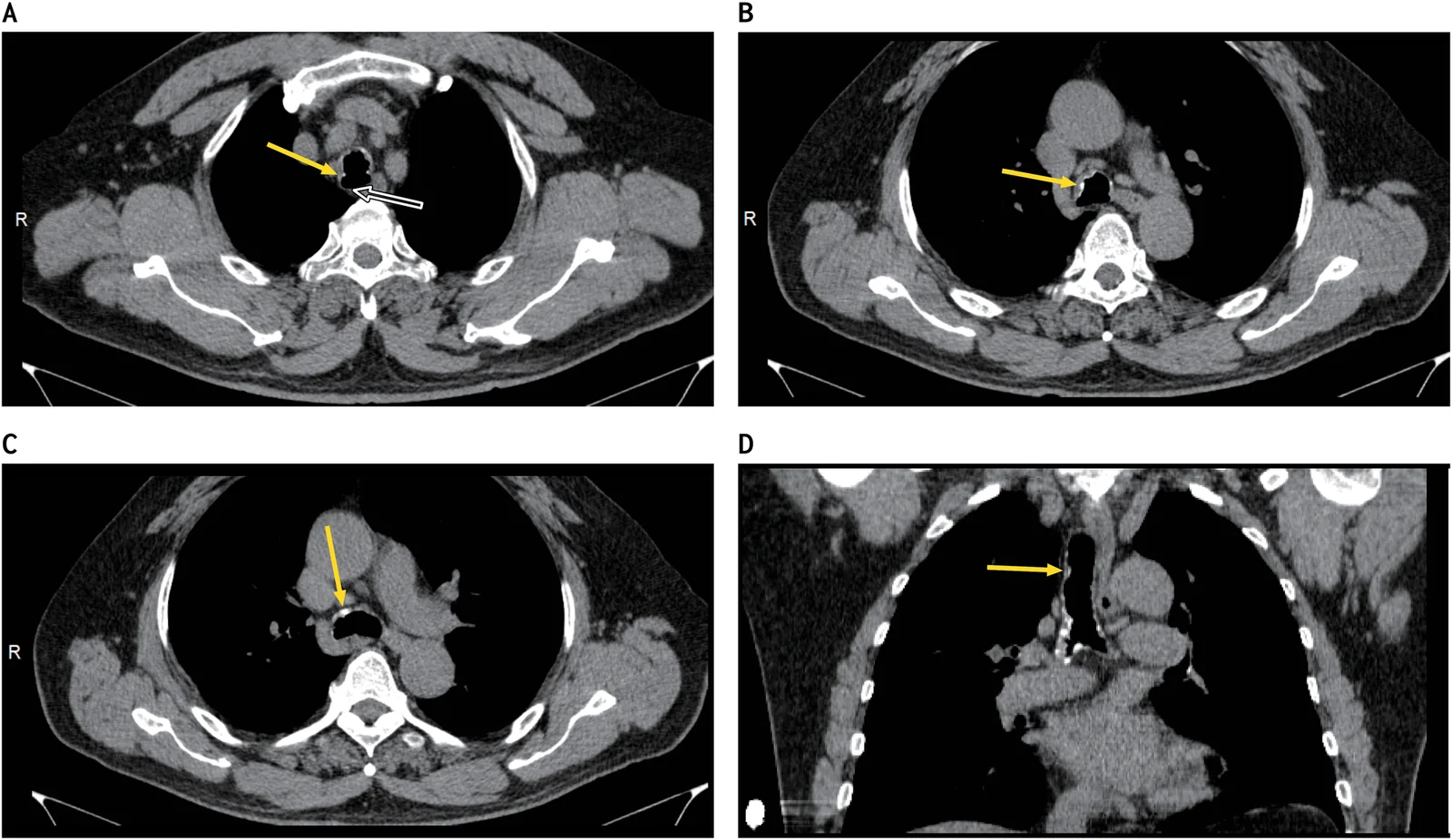
Chest CT findings of tracheobronchopathia osteochondroplastica. **(A)** Axial image at the upper-to-mid tracheal level shows partially calcified anterolateral nodular protrusions (yellow arrow), with relative sparing of the posterior membranous wall (open white arrow). **(B)** Axial image at the lower tracheal level shows irregular nodular wall thickening with intraluminal protrusion (yellow arrow). **(C)** Axial image near the carina shows distal tracheal nodular thickening (yellow arrow). **(D)** Coronal multiplanar reformation shows longitudinal involvement of the trachea and proximal bronchi, with a representative tracheal lesion indicated by the yellow arrow.

Pulmonary function testing on 8 June 2026 showed a forced vital capacity of 3.50 L (92% predicted), forced expiratory volume in 1 s of 2.67 L (89% predicted), and FEV₁/FVC of 76.4%. Total lung capacity was 6.20 L (95% predicted) and residual volume 2.61 L (110% predicted). The available flow-volume tracing did not show the classic box-shaped flattening of both limbs associated with fixed central-airway obstruction (Supplementary Figure S1). Methacholine challenge testing was negative. Diffusing capacity for carbon monoxide (DLCO) was 3.99 mmol·min⁻^1^·kPa⁻^1^ (46% predicted), with a transfer coefficient of 0.66 mmol·min⁻^1^·kPa⁻^1^·L⁻^1^ (50% predicted) and alveolar volume of 6.01 L (95% predicted). Detailed test-quality indices and hemoglobin-correction information were unavailable, limiting interpretation of the reduced DLCO.

Flexible bronchoscopy on 8 June 2026 revealed hard yellow-white nodules along the anterior and lateral tracheal walls, with partial confluence and relative posterior-wall sparing. Similar nodules involved the carina, left main bronchus, right main bronchus, and bronchus intermedius. The right middle lobe bronchial orifice was narrowed, and increased mucoid secretions were seen in the right upper lobe bronchus. Five biopsies were taken from anterolateral tracheal nodules. Bronchial brushing and bilateral BAL were performed, with lavage fluid pooled into one specimen (Figure 2). Sampling occurred after empirical piperacillin/tazobactam had been initiated that day. The bronchoscopic impression was TO with concomitant mucosal inflammation.

**Figure 2:**
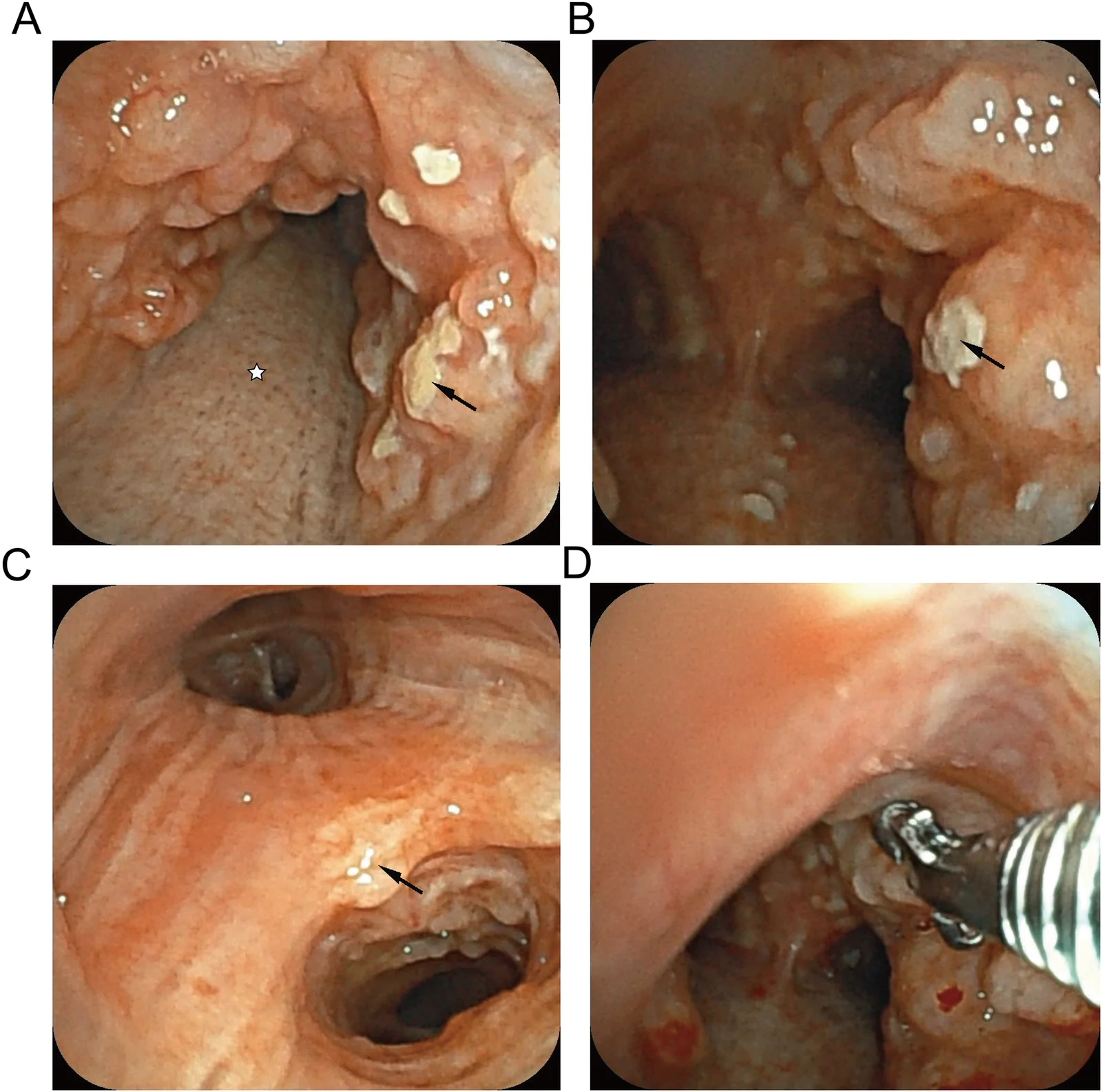
Bronchoscopic findings of tracheobronchopathia osteochondroplastica. **(A)** Hard yellow-white nodules involve the anterior and lateral tracheal walls (black arrow), with relative sparing of the posterior membranous wall (white asterisk). **(B)** Close-up view shows partially confluent nodules with a cobblestone-like appearance (black arrow). **(C)** Similar nodules at a proximal bronchial bifurcation cause focal luminal irregularity (black arrow). **(D)** Forceps biopsy of a tracheal nodule.

The pooled BAL culture, reported on 11 June 2026, yielded *P. aeruginosa* at >10⁴ CFU/mL, susceptible to all tested antipseudomonal agents, including piperacillin/tazobactam and levofloxacin. Acid-fast staining and liquid mycobacterial culture of bronchoscopic lavage material were negative, as were fungal smear and fungal culture. Galactomannan and *β*-D-glucan assays were not performed. Cytology showed predominantly ciliated epithelial cells with a small number of macrophages and neutrophils. No sputum or other lower-airway culture was repeated after treatment.

Tracheal biopsy showed fragments of respiratory mucosa with stromal hemorrhage and sparse inflammatory cells, together with mature lamellar bone containing osteocytes within lacunae (Figure 3). These findings supported TO in the context of the characteristic CT and bronchoscopic appearances. Respiratory symptoms, a pulmonary opacity, and recovery of *P. aeruginosa* supported a clinically suspected concomitant lower respiratory tract infection, although the organism's role could not be distinguished conclusively from airway colonization.

**Figure 3:**
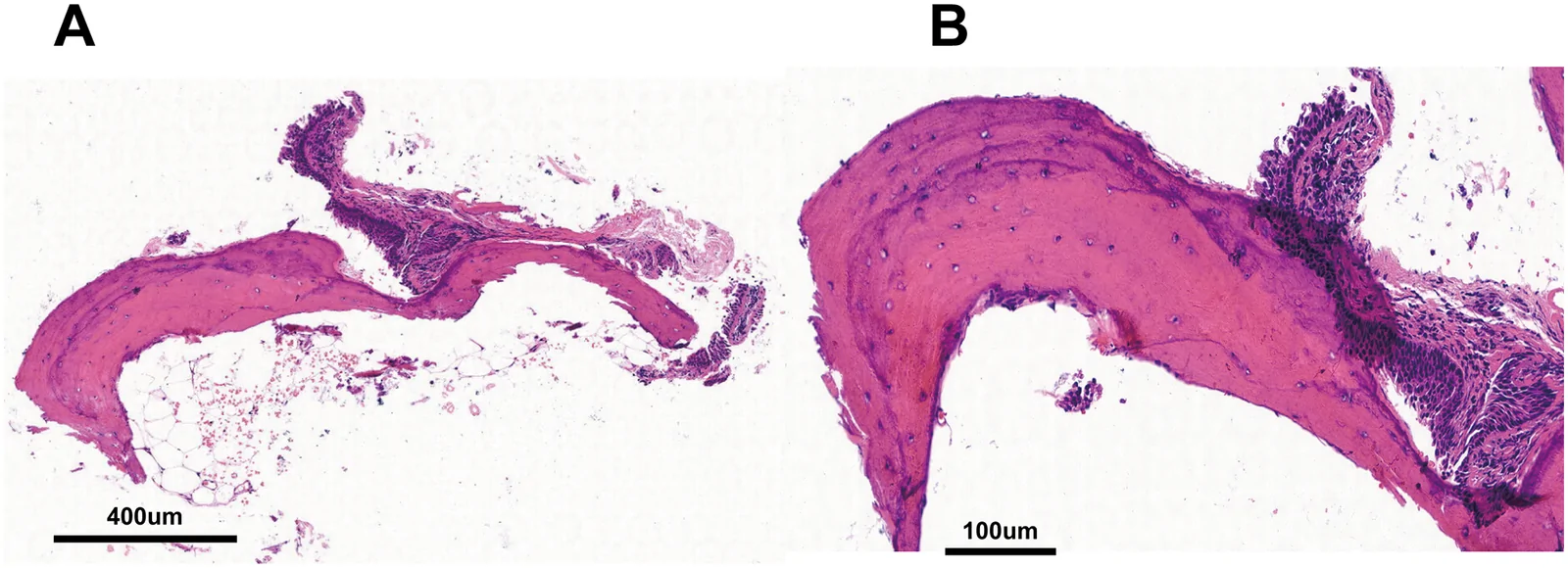
Histopathological findings of the tracheal biopsy. **(A)** Low-power hematoxylin and eosin (H&E)-stained section shows fragments of respiratory mucosa adjacent to a focus of mature osseous tissue. **(B)** Higher-power H&E-stained section demonstrates mature lamellar bone with osteocytes within lacunae. Scale bars, 400 μm **(A)** and 100 μm **(B).**

### 2.3 Therapeutic intervention and follow-up

Piperacillin/tazobactam was started empirically on 8 June 2026 for suspected lower respiratory tract infection, before the culture result became available. The documented regimen was 4.5 g intravenously every 12 h, each dose infused over approximately 1 h, through 12 June inclusive (5 days). The susceptibility result on 11 June confirmed *in vitro* activity of the ongoing treatment. After clinical improvement, the patient was discharged on 12 June and completed oral levofloxacin 500 mg once daily for 9 days, giving a documented 14-day antimicrobial course. He reported completing the oral course without adverse effects. Additional treatment comprised ambroxol, nebulized budesonide and terbutaline, and airway-clearance measures. Bronchoscopic intervention was deferred because there was no clinical evidence of central-airway obstruction requiring treatment.

At telephone follow-up 2 months after discharge, he reported no significant recurrent respiratory symptoms. At an outpatient visit on 19 September 2026, 99 days after discharge, he was asymptomatic. Comparison of follow-up CT with baseline images by the treating clinician showed resolution of the previous pulmonary opacity, no obvious change in the tracheobronchial nodules, and stability of the individually reviewed ground-glass and solid pulmonary nodules. These were the treating clinician's observations; a formal radiology comparison report was unavailable. Follow-up bronchoscopy and repeat cultures were not performed. Table 1 summarizes the clinical course

**Table 1:** Timeline of the clinical course.

**Date**	**Clinical event**	**Main findings and management**
Approximately March 2026	Symptom onset	Cough with scant, difficult-to-expectorate whitish mucoid sputum.
Before admission	Local treatment	Unspecified intravenous treatment with limited benefit; antibiotic exposure, names, and duration could not be verified.
30 May 2026	Baseline chest CT	Right-sided pulmonary opacity, ground-glass and solid nodules, and partially calcified anterolateral tracheobronchial nodules. Retrospective minimum tracheal area 179.4 mm^2^ and Dmin 10.0 mm.
8 June 2026	Admission and treatment initiation	Stable vital signs with persistent cough and sputum. Empirical piperacillin/tazobactam started before culture results.
8 June 2026	Bronchoscopy and sampling	Hard tracheobronchial nodules with posterior-wall sparing and focal bronchial narrowing. Biopsies and pooled bilateral BAL obtained after antibiotic initiation; biopsy subsequently showed mature lamellar bone.
11 June 2026	BAL culture reported	*P. aeruginosa* >10⁴ CFU/mL, susceptible to piperacillin/tazobactam and levofloxacin.
8–12 June 2026	Intravenous treatment	Piperacillin/tazobactam 4.5 g every 12 h, infused over approximately 1 h per dose, for 5 days; symptomatic treatment and airway clearance.
12 June 2026	Discharge and oral treatment	Symptoms improved. Completed levofloxacin 500 mg once daily for 9 days; total documented antimicrobial course 14 days. No reported adverse effects.
2 months after discharge	Telephone follow-up	No significant recurrent respiratory symptoms.
19 September 2026	Outpatient and CT follow-up	Asymptomatic. Treating clinician's CT comparison showed resolution of the pulmonary opacity, unchanged airway nodules, and stable pulmonary nodules. No formal radiology comparison report, repeat bronchoscopy, or repeat culture available.

## 3 Discussion

### 3.1 Epidemiology and clinical presentation

Consistent with published series, the present patient was diagnosed in later adulthood and presented with chronic cough and sputum production rather than symptoms specific to central-airway disease (1–6). The absence of wheeze, hemoptysis, or clinically significant dyspnea further illustrates why TO may remain unrecognized until CT or bronchoscopy is performed for another indication.

### 3.2 Potential pathogenesis

The pathogenesis of TO remains unresolved. Chronic mechanical or inflammatory injury has been proposed to induce metaplastic transformation of submucosal connective tissue, ectopic cartilage formation, and subsequent ossification. Tajima et al. identified bone morphogenetic protein-2 and transforming growth factor-*β*1 expression within TO lesions, supporting a role for osteochondrogenic signaling (10). Hong et al. subsequently demonstrated dysregulated airway basal stem-cell function and inflammatory/TGF-*β*/BMP signaling, providing a potential mechanistic link between epithelial metaplasia and mesenchymal osteochondrogenesis (11). These findings are biologically plausible but do not establish causation in an individual patient.

### 3.3 Integrated diagnostic interpretation

TO is recognized through concordant CT and bronchoscopic findings. Typical features include partially calcified, irregular nodular thickening of the anterolateral tracheobronchial walls and hard submucosal nodules with relative posterior-wall sparing (2, 3, 7–9). The present case showed this distribution from the trachea to the proximal bronchi, including narrowing of the right middle lobe bronchial orifice. The minimum tracheal luminal area of 179.4 mm^2^ and corresponding Dmin of 10.0 mm document airway caliber without assigning a percentage stenosis against an abnormal reference segment. Anatomic narrowing should be interpreted together with symptoms and functional findings when considering intervention. Histopathologic examination may reveal cartilage, calcification, or mature bone. Although some case series suggest that biopsy may be unnecessary when CT and bronchoscopic findings are classic (3, 8), Nair et al. identified alternative or coexisting etiologies in 38% of biopsied patients and advocated tissue sampling, when feasible, to exclude clinically important mimics (9). Biopsy is particularly valuable when the lesion distribution is atypical, the posterior membranous wall is involved, or amyloidosis, papillomatosis, inflammatory stenosis, or malignancy is suspected. In the present patient, the small, fragmented biopsy specimen demonstrated mature lamellar bone with osteocytes within lacunae, closely corresponding to the radiologic and bronchoscopic phenotype. However, because the specimen did not show the complete architecture of a large osteocartilaginous nodule, the histopathologic findings were interpreted as supportive rather than independently diagnostic.

### 3.4 Differential diagnosis

Important mimics include tracheobronchial amyloidosis, relapsing polychondritis, granulomatosis with polyangiitis, tuberculosis, papillomatosis, and neoplastic airway disease (7, 9). Amyloidosis can cause calcified nodules but often involves the posterior wall; Congo red staining is diagnostic when amyloid is demonstrated. Relapsing polychondritis more often produces smooth cartilaginous wall thickening, destruction, or collapse with systemic cartilage involvement. Granulomatosis with polyangiitis and tuberculosis may produce inflammatory, ulcerative, or cicatricial stenosis; papillomatosis produces papillary lesions. Hard anterolateral nodules, partial calcification, posterior-wall sparing, negative mycobacterial studies, and mature bone on biopsy collectively favored TO in this patient.

Fungal airway disease, relevant inhalational exposures, and immunodeficiency were also considered. Fungal smear and culture of bronchoscopic lavage material were negative, and CT showed no cavitation. However, galactomannan and *β*-D-glucan were not tested, so fungal disease was not comprehensively excluded. Focused history found no asbestos, crystalline silica, stone-processing, or construction exposure. HIV antibody testing was negative and no prolonged immunosuppressive treatment was reported; unmeasured immunoglobulin concentrations precluded exclusion of a humoral immune defect. These findings reduced concern for particular alternatives without establishing normal immune function.

### 3.5 TO and recovery of *Pseudomonas aeruginosa*

Airway irregularity and focal narrowing in TO may impair secretion clearance, providing a plausible substrate for respiratory infection; this mechanism was not measured in our patient. *P. aeruginosa* has previously been recovered from lower-airway samples in TO (12, 13), so coexistence alone is not novel. Here, respiratory symptoms, a patchy pulmonary opacity, quantitative BAL recovery, *in vitro* antimicrobial susceptibility, and subsequent clinical and radiologic improvement supported a clinically suspected concomitant lower respiratory tract infection. Persistence of airway nodules despite resolution of the pulmonary opacity helped distinguish structural TO from the reversible pulmonary process.

The etiologic interpretation remains limited. The patient was afebrile, had no leukocytosis, and lacked admission CRP/procalcitonin measurements. Mucoid secretions and a culture result alone do not establish pneumonia due to *P. aeruginosa*. Bilaterally pooled BAL prevented localization of bacterial recovery to the right-sided opacity or a particular narrowed bronchus. Sampling after antibiotics had begun, together with uncertainty about previous intravenous treatment, may have affected culture yield. No repeat culture documented clearance, and later radiologic improvement was not pathogen-specific. CT showed no bronchiectasis, emphysema, mucus plugging, atelectasis, or other listed parenchymal risk features, but their absence does not prove a causal role for TO. Colonization accompanying an independently resolving pulmonary infection remained possible. We therefore report *P. aeruginosa* recovered from BAL during a suspected respiratory infection, without claiming TO-induced *Pseudomonas* pneumonia.

### 3.6 Pulmonary function and reduced diffusing capacity

Preserved spirometry does not exclude structural TO (14). The available flow-volume tracing lacked a classic fixed-obstruction pattern, but this and preserved FEV₁/FVC do not exclude every form of small-airway or dynamic central-airway dysfunction. Accordingly, the findings support the absence of evident obstruction requiring intervention rather than a categorical exclusion of obstructive lung disease. DLCO remained unexplained despite preserved alveolar volume. CT showed no emphysema or interstitial lung disease, but unavailable test-quality and hemoglobin-correction data limited interpretation. The reduced DLCO was not attributed to TO; reassessment would be appropriate if clinically indicated.

### 3.7 Management and follow-up

No disease-modifying therapy for TO has been established. Asymptomatic or mildly symptomatic patients are generally managed conservatively with observation, airway humidification and clearance, expectorant therapy, treatment of coexisting infection, and management of relevant comorbidities (3, 6, 9). In patients with clinically significant central-airway obstruction, persistent dyspnea, recurrent post-obstructive infection, or major airway-management difficulty, individualized bronchoscopic treatment may include mechanical debulking, laser or argon plasma coagulation, cryotherapy, balloon dilation, or stent placement; however, the evidence is derived mainly from case reports and small series.

The absence of clinically significant dyspnea, preserved spirometric indices, and favorable clinical course supported conservative airway management. Antimicrobials and supportive treatment were followed by symptomatic improvement and later resolution of the pulmonary opacity, while the airway lesions remained unchanged on the treating clinician's CT review. This short-term course is compatible with reports of stable TO (3, 6, 15). but approximately 3 months of clinical follow-up cannot establish long-term stability. Continued surveillance of both airway disease and the separate pulmonary nodules remains necessary. Recurrent focal infection, atelectasis, progressive dyspnea, or declining airflow would prompt reassessment of the need for bronchoscopic intervention.

### 3.8 Strengths and limitations

This case links CT, bronchoscopic, and supportive histopathologic findings with quantified airway caliber and clinical and imaging follow-up. It demonstrates the practical value of distinguishing a resolving pulmonary opacity from persistent airway nodules and separate pulmonary nodules. Limitations include a small fragmented biopsy, absent admission inflammatory biomarkers, BAL obtained after antibiotics and pooled bilaterally, unknown preceding antibiotic exposure, and no microbiologic reassessment. Fungal and immune evaluations were incomplete. Follow-up lasted approximately 3 months after discharge, repeat bronchoscopy was unavailable, and CT comparison reflected the treating clinician's assessment without a formal radiology comparison report. Short-term stability does not establish benignity of the pulmonary nodules. The lack of a normal tracheal reference prevented calculation of percentage stenosis, although absolute luminal area and diameter were measured. Reduced DLCO remained unexplained. These limitations prevent proof that *P. aeruginosa* caused the pulmonary opacity or that TO predisposed this individual to infection.

## 4 Conclusion

TO should be considered when CT and bronchoscopy show concordant partially calcified anterolateral airway nodules with relative posterior-wall sparing; limited biopsy findings can provide support. Clinical and radiologic follow-up can separate persistent airway abnormalities from a resolving concomitant pulmonary process. Recovery of *P. aeruginosa* from BAL requires cautious interpretation and does not establish either organism-specific pneumonia or causation by TO. Conservative management and continued surveillance should reflect symptoms, functional impact, and associated pulmonary findings.

## 5 Patient perspective

At the 2-month telephone follow-up, the patient expressed the following perspective: “After treatment, my cough and sputum production improved markedly. The physicians explained that the nodules in my trachea and bronchi represented a rare but benign condition and that the airway narrowing was not severe enough to require an interventional procedure at present. At the 2-month telephone follow-up, I had no significant recurrent respiratory symptoms. I am willing to continue follow-up as advised.”

## Data Availability

The original contributions presented in this case report are included in the article and Supplementary Material. Further inquiries can be directed to the corresponding author.
