# Supplementary material for: Case Report: Tracheobronchopathia osteochondroplastica with Pseudomonas aeruginosa recovered from bronchoalveolar lavage fluid
Source: Front Med (Lausanne). 2026 Oct 21;13:1972198. doi: 10.3389/fmed.2026.1972198 (PMC13731277; doi:10.3389/fmed.2026.1972198)
Supplement: Supplementary file 1 [file Datasheet1.docx]

Supplementary material

Case Report: Tracheobronchopathia osteochondroplastica with *Pseudomonas aeruginosa* recovered from bronchoalveolar lavage fluid

# Supplementary Figure S1

Supplementary Figure S1. Flow-volume loop from the pulmonary function report obtained on 8 June 2026. The original tracing is reproduced without redrawing the recorded curves; patient identifiers and unrelated report elements have been removed by cropping. The horizontal axis represents volume in liters and the vertical axis flow in liters per second. Positive flow denotes expiration and negative flow inspiration. The tracing lacks the classic box-shaped flattening of both limbs associated with fixed central-airway obstruction. This observation does not exclude all central-airway or small-airway dysfunction, and detailed maneuver acceptability and repeatability data were unavailable.
